# Non-pharmacological interventions for the prevention of sexually transmitted infections (STIs) in older adults: A systematic review

**DOI:** 10.1371/journal.pone.0284324

**Published:** 2023-05-24

**Authors:** Melissa Co, Darío Moreno-Agostino, Yu-Tzu Wu, Elyse Couch, Ana Posarac, Teodora Wi, Ritu Sadana, Sophie Carlisle, Matthew Prina

**Affiliations:** 1 Health Service and Population Research Department, Institute of Psychiatry, Psychology and Neuroscience, King’s College London, London, United Kingdom; 2 Centre for Longitudinal Studies, UCL Social Research Institute, University College London, London, United Kingdom; 3 ESRC Centre for Society and Mental Health, King’s College London, London, United Kingdom; 4 Population Health Sciences Institute, Faculty of Medical Sciences, Newcastle University, Newcastle upon Tyne, United Kingdom; 5 Center for Gerontology and Healthcare Research, Brown University, Providence, RI, United States of America; 6 Ageing and Health Unit, Maternal, Newborn, Child and Adolescent Health and Ageing Department, World Health Organisation, Geneva, Switzerland; 7 Department of Global HIV, Hepatitis and Sexually Transmitted Infections Programmes, World Health Organisation, Geneva, Switzerland; 8 World Health Organization Secretariat, Council on the Economics of Health for All, Geneva, Switzerland; University of Brighton, UNITED KINGDOM

## Abstract

**Background:**

STIs in older adults (adults aged 50 years and older) are on the rise due to variable levels of sex literacy and misperceived susceptibility to infections, among other factors. We systematically reviewed evidence on the effect of non-pharmacological interventions for the primary prevention of sexually transmitted infections (STIs) and high-risk sexual behaviour in older adults.

**Methods:**

We searched EMBASE, MEDLINE, PSYCINFO, Global Health and the Cochrane Library from inception until March 9^th^, 2022. We included RCTs, cluster-randomised trials, quasi-RCTs, interrupted time series (ITS) and controlled and uncontrolled before-and-after studies of non-pharmacological primary prevention interventions (e.g. educational and behaviour change interventions) in older adults, reporting either qualitative or quantitative findings. At least two review authors independently assessed the eligibility of articles and extracted data on main characteristics, risk of bias and study findings. Narrative synthesis was performed.

**Results:**

Ten studies (two RCTs, seven quasi-experiment studies and one qualitative study) were found to be eligible for this review. These interventions were mainly information, education and communication activities (IECs) aimed at fostering participants’ knowledge on STIs and safer sex, mostly focused on HIV. Most studies used self-reported outcomes measuring knowledge and behaviour change related to HIV, STIs and safer sex. Studies generally reported an increase in STI/HIV knowledge. However, risk of bias was high or critical across all studies.

**Conclusions:**

Literature on non-pharmacological interventions for older adults is sparse, particularly outside the US and for STIs other than HIV. There is evidence that IECs may improve short-term knowledge about STIs however, it is not clear this translates into long-term improvement or behaviour change as all studies included in this review had follow-up times of 3 months or less. More robust and higher-quality studies are needed in order to confirm the effectiveness of non-pharmacological primary prevention interventions for reducing STIs in the older adult population.

## Introduction

The increasing prevalence of sexually transmitted infections (STIs) among older adults—a term we will use in this study to refer to people aged 50 and over—is a growing public health concern [1, 2]. STIs encompass many conditions, including chlamydia, gonorrhoea, hepatitis B, herpes simplex virus (HSV), human immunodeficiency virus (HIV), human papilloma virus (HPV), syphilis and trichomoniasis [3]. Although these conditions are more commonly diagnosed in younger age groups, a review which summarised evidence from the US, Canada, UK, China and Africa has shown increasing trends in the diagnostic rates of STIs among older adults [1]. Calculations based on data from the UK Health Security Agency indicate that between 2016 and 2019 there was an 10% increase in new cases of STIs in people aged 13–64 in England, in contrast to an increase of 31% among adults aged 65 and over [4]. An analysis of STI surveillance data in the UK has also shown a doubling of new infections from chlamydia, genital herpes and warts, gonorrhoea and syphilis from 1996 to 2003 in people over 45 [5]. In the US, people aged 50 and over comprised 51% of those living with an HIV diagnosis in 2018 [6], partly but not exclusively due to longer survival due to treatment [7], and high STI prevalence estimates have been reported more broadly in older adults across the globe, including China, Korea, Kenya and Botswana [8–11].

While the prevalence of STIs among older adults is growing, this still equates to a small number of people and a small proportion of the total STI burden. For example, in the UK 37,692 new STI diagnoses were reported in persons over 45 in 2019, representing 8% of all new diagnoses made that year [12]. Similarly, out of 36,801 people newly diagnosed with HIV in the US in 2019, 3,887 (10.6%) were over the age of 55 [13]. However, a survey of attenders in three genitourinary medicine clinics in the UK suggests that older people are less likely to seek treatment for STIs and have longer delays between symptom recognition and health care presentation compared to younger individuals [14]. They are also less likely to receive routine STI testing due to healthcare personnels’ misconceptions about the sexuality and sexual activity of older adults [15]. Raising awareness about the importance of routine STI testing among older adults themselves may also reduce the risk of further spread of infection, as the perception of low risk of infection may be a barrier to seeking testing [16]. If left untreated, STIs may be transmitted to sexual partners, leading to medical complications and contributing to the burden of multimorbidity, often seen among older people [17].

The rise in STIs among older adults might be attributed to multiple factors. For example, the increase in HIV cases has been suggested to be the result of longer survival due to treatment, inconsistent use of condoms, misperceived susceptibility to infection, and variable levels of sexual health knowledge [7, 18]. Just as in other life stages, older adults may engage in high-risk sexual behaviours due to lack of knowledge or misconceptions on the risks involved. For instance, heterosexual adults that have undergone menopause or sterilisation/vasectomy may feel less motivated to use condoms as pregnancy may no longer be a concern [19]. Primary prevention measures focused on behaviour change communication—using tailored communications approaches to promote positive behaviours, ideally involving the target community in the development process [20]—are therefore key to reducing the spread of STIs. Increasing older adults’ knowledge of the risk of STIs, how to prevent them, and how to engage in healthy sexual behaviours through behaviour change interventions could reduce their odds of engaging in high-risk sexual behaviours relative to protective ones, thus subsequently reducing STI risk in older age [18].

There are currently a number of existing systematic reviews in the broader area of STI prevention but they: 1) do not focus on older adults; 2) only focus on specific STIs or secondary prevention; 3) only focus on specific type of prevention (i.e. condom use). Ward and colleagues published a review on the effectiveness of behavioural interventions on the reduction of STI among genitourinary medicine clinical patients [21]. Although most studies showed a greater reduction in STI rates and self-reported sexual behaviour in intervention groups, compared to control groups, none of the studies included focused exclusively on older adults. Three systematic reviews focused specifically on educational and behavioural prevention of HIV in older adults [7, 22, 23] and were published between 2012 and 2014. All the reviews included both primary and secondary prevention (i.e. studies including people living with HIV), only focused on HIV and highlighted that more evidence is needed to understand the effectiveness of non-pharmacological primary prevention interventions in older adults. One review focused on interventions to increase condom use among middle-aged and older adults [24]. Although five interventions were included, the studies only recruited people living with HIV or focused on middle-aged individuals. Finally, three reviews focused specifically on digital interventions for the prevention of STIs, however of these three, two focused on HIV only, none focused specifically on older adults [25–27]. There is therefore a need for a broader and up-to-date systematic review of non-pharmacological primary prevention interventions of STIs among older adults.

We therefore aimed to review the literature and identify studies that investigated the effect of non-pharmacological interventions (such as behaviour change interventions) for the prevention of sexually transmitted infections (STIs) and high-risk sexual behaviour in older adults.

## Methods

This review has been prepared following the Preferred Reporting Items for a Systematic Review and Meta-Analysis (PRISMA) [28], with its checklist readable in S1 Appendix. A pre-registered protocol is available on PROSPERO under registration number CRD42020177457.

The following criteria were used for considering studies for this review:

### Types of studies

We expected to see a variety of approaches to evaluating interventions. These could include randomised controlled trials (RCTs), cluster-randomised trials, quasi-RCTs, interrupted time series (ITS), controlled before-and-after studies (CBA) and uncontrolled before and after studies. Studies reporting either qualitative or quantitative findings were included.

### Types of participants

We included interventions targeting adults aged 50 and over without known STIs (primary prevention). Studies including both younger and older adults were only included if more than 50% of the participants were 50 and over, or if findings were presented separately for the older age group. Studies where the age of the participants was unknown were excluded. Additionally, studies focussed on health providers were excluded as this was not the target population of the interventions.

### Types of interventions

Broadly, intervention objectives needed to relate to reducing STIs or risky sexual behaviours or promoting awareness/knowledge around safer sex and STI prevention. We included studies of any non-pharmacological primary prevention activities, expecting mainly behaviour change communication interventions with the goal of changing cognitive outcomes such as participants’ knowledge or beliefs, behavioural outcomes, or biological outcomes such as the reduction of STIs. Multicomponent interventions were included as long as there was not a pharmaceutical component. Interventions could focus on a range of issues, including information about STIs and their course, protective versus risky sexual behaviours, condom use, the importance of routine STI testing or vaccinations, mutual monogamy, abstinence and more [29]. We included studies on interventions done via a range of media, such as (but not limited to) individual or group meetings, informational videos, written documents (such as paper or digital leaflets), text messages, phone calls, television or poster campaigns, or combinations of these. We included studies using interventions specifically tailored to older adults as well as studies generally measuring effectiveness of non-pharmacological primary prevention interventions in this age group. We hoped this broader inclusion criteria would help to capture as much evidence as possible for non-pharmacological primary prevention interventions in older adults. Interventions aimed at healthcare practitioners were excluded.

### Types of comparisons

We did not include or exclude studies based on type of control group, as we expected a wide variety of study types which might be controlled or uncontrolled. As this area is particularly under-studied, we hoped that this would maximise comprehensiveness of the review.

### Types of outcome measures

We also did not include or exclude based on specific outcome measures, as we expected studies to report a wide range of outcomes and wanted to capture as many relevant studies as possible. Examples of possible outcomes we expected include:

- Biological
  ○ Incidence of STI.
  ○ Detection rate of STI.
- Behavioural
  ○ Self-reported use of condoms.
  ○ Self-reported unprotected intercourse.
  ○ STI health seeking behaviour. (e.g., uptake of STI testing)
  ○ Uptake of STI services, (e.g., use of STI clinics).
- Cognitive
  ○ Increase in knowledge of STIs or safer sex practices.

Qualitative data were also extracted and analysed to identify the barriers and facilitators to the successful implementation of non-pharmacological interventions to prevent STIs.

### Types of publication

Reports of peer-reviewed, primary research was included. Non-primary reports, such as editorials and secondary research such as reviews were excluded. Conference abstracts were also excluded as these are not necessarily peer-reviewed and are unlikely to contain all necessary information.

### Search methods for identification of studies

A search strategy was carried out in EMBASE, MEDLINE, PSYCINFO, Global Health and the Cochrane Library from inception until March 9^th^, 2022 (an initial search was conducted on March 27^th^, 2020 and later updated). The full search strategy is available in S2 Appendix. We also hand-searched relevant articles and reviews on the topic and screened the reference lists of included studies.

### Selection of studies

All search results were merged into Endnote and deduplicated. Titles, abstracts and subsequently full texts were double-screened independently in the initial search and additional articles were screened by one author in the updated search (MC, DMA, MP, YTW, EC, SC) using Rayyan QCRI [30]. Disagreements were resolved through discussion, occasionally involving a third screener.

### Data extraction and management

Data was extracted from each study using a standardised data extraction form in Excel. The form included the following information:

- General study characteristics: study name, country, setting, funding information, years study active, study design, length of follow-up period, study population, selection of control/comparison group, selection of intervention group.
- Intervention characteristics: mode of delivery, setting of delivery, target population for intervention, specific components of interventions.
- Participant characteristics: age range, average age, gender, number in control and intervention groups.
- Study results: definition and measurement of outcomes in intervention and control (or pre- and post-) groups, statistical difference between intervention and control groups, any additional subgroup analyses performed, study authors’ overall assessment of intervention effectiveness and qualitative data.

All data included in this review are available from the articles cited here.

### Assessment of risk of bias in included studies

Two authors independently assessed risk of bias for each study. Any disagreement in ratings was discussed and a consensus reached. We followed guidance from the Cochrane Handbook for Systematic Reviews of Interventions [31]. For RCTs, we used the Cochrane Risk of Bias tool for randomised trials (RoB 2) [32]. Non-randomised and non-controlled studies were assessed using the Cochrane Risk of Bias in Non-Randomized Studies of—Interventions (ROBINS-I) [33]. The ROBVIS tool (https://mcguinlu.shinyapps.io/robvis/) was used to visualise the risk of bias in our systematic review [34]. Qualitative studies were assessed using the Joanna Briggs Critical Appraisal Tool for qualitative research [35].

### Data synthesis

Due to the heterogeneity in methods, designs, interventions, and outcomes, we felt that a meta-analysis would not yield meaningful results for informing future interventions. Narrative synthesis was used to summarise findings for all included studies, following Popay et al.’s guidance [36]. Study data (study characteristics, participant characteristics, intervention characteristics, results) were tabulated and are presented.

We used thematic synthesis to analyse qualitative findings reported by the included studies [37]. We were particularly interested in drawing out themes related to the barriers and facilitators of sex education interventions for older adults. The relevant passages of the included studies were coded line-by-line, and similar codes were then grouped into descriptive themes which described the contents of the included papers. The descriptive themes were further refined into analytical themes related to the aim of the synthesis. The analytical themes were cross-checked by two researchers against the initial codes to ensure consistency between the findings of our synthesis and the evidence presented in the included papers.

## Results

### Literature search

**Fig 1:**
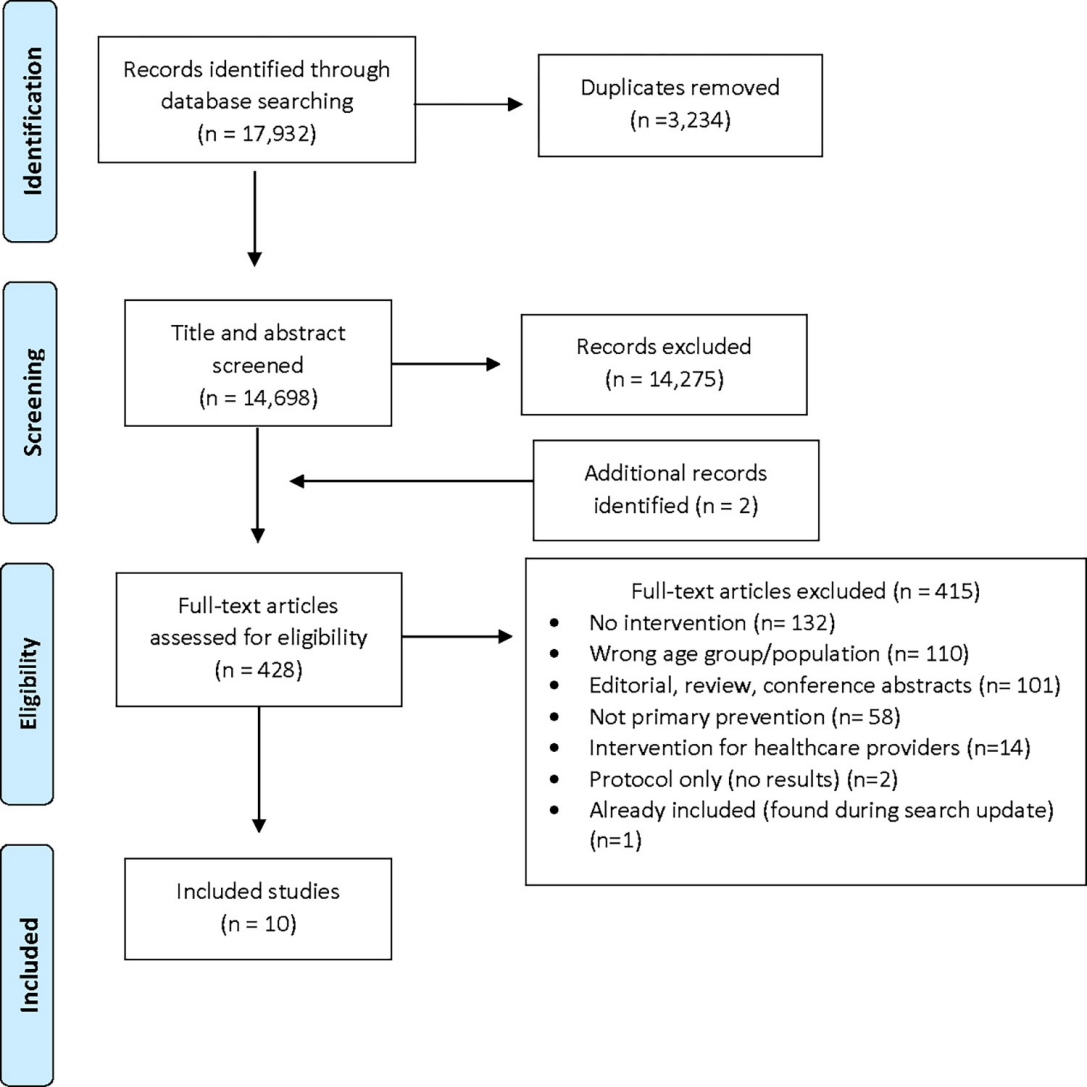
Flowchart of literature search.

The literature search identified 17,932 records. After removing duplicates, 14,275 were excluded in the title and abstract screening stage, and an additional two articles identified. The full text review was carried out for 425 publications and 415 were excluded for these reasons: did not include a sexual health intervention (n = 132), wrong age group (younger or unknown age of the study population; n = 110), editorial, review or conference abstracts (n = 101), not primary prevention (n = 58) and intervention for health providers (n = 14). Ten publications were found to be eligible for this review (Fig 1).

### Characteristics of included studies

Table 1 reports characteristics of included studies. Among the ten included studies, two were RCTs [38, 39], seven were quasi-experimental pre-post studies [40–46], one was a qualitative study [18], and two reported additional qualitative findings [43, 46]. The majority of studies were based in the US and only two recent studies were from China [38] and South Africa [40]. Apart from the two clinical trials, all studies were carried out nearly ten years ago.

**Table 1:** Characteristics of included studies (n = 10).

Study			Participants			Intervention/ Control	Outcomes			Analysis
First author, year	Type of intervention	Country, study year	N	Description	Mean age; sex (%)	Content	Content	Time points measured	Sample for analysis (% drop-out/ missing)	Strategy and statistical methods
**Randomised control trials**										
Liu, 2019	IEC	China, 2017–18	300	Community-based men (aged 50+) who had non-marital sex	65.6; men 100%	Intervention: IECs, demonstration and consulting (four sessions over 1 year): (1) 1 to 1 HIV education; (2) Sharing peer studies and providing consulting service; (3) 1 to 1 Demonstration of condom use; (4) Expert lecture Control: no intervention	Self-reported (1) Incidence of non-marital sex; (2) Incidence of commercial exchange of sex for drug, money, gift; (3) percentage of condom use	3 months after intervention	284 (5%)	Naïve per-protocol analysis; Chi-sq. test
Weitzman, 2019	Website	USA, Unknown	331	Divorced or separated women (aged 50+)	54.6; women 100%	Intervention: Website materials for HIV/STD and safer sex education; twice weekly emails/texts Control: Emails with written materials for HIV/STD and safer sex education	Self-reported intention to practice safer sex (a three-item intention to engage in safer sex practices instrument); pre-post differences	60 days after intervention	219 (34%)	Intention to treat and complete case analysis; Linear regression adjusted for age, ethnicity, income depression and location of recruitment
**Quasi-experiment, pre-post**										
Boon, 2009	IEC	South Africa, Unknown	202	isiXhosa speaking people (aged 60+) who took care of their sick children or grandchildren with HIV	-	Intervention: Four workshops (3-hour sessions for 4 weeks): (1) HIV information; (2) intergenerational communication skills; (3) home-based nursing care; (4) Social assistance and available support Control: NA	Self-reported 10 items on HIV knowledge	4 weeks for pre-post, 3 months for follow-up	154 (24%)	Complete case analysis; ANCOVA
Falvo, 2004	IEC	USA, Unknown	40	Volunteers from retirement homes and senior centres in Ohio	73.8; women 87.5%	Intervention: One workshop (45 mins) on safer sex practices and HIV education Control: NA	Self-reported, modified AIDS Knowledge Survey, modified Bobowicz Sex Inventory	3 months after intervention (up to 8 months for mail-in responses)	34 (15%)	Complete case analysis; Paired sample t-tests comparing pre-, post-, follow-up
Orel, 2010	IEC	USA, 2010	89	Individuals preregistered for the workshop (n = 36) and older adults who attended the educational components of the conference (n = 53)	75; women 66%	Intervention: One workshop (1 day) with three sessions: (1) sexuality in middle and later adulthood; (2) HIV and other STIs; (3) medication and sexuality. Control: NA	Self-reported 45-item HIV Knowledge Questionnaire	Immediate after intervention	11 (88%)	None
Reisner, 2011	IEC	USA, 2008–11	97	Older gay and bisexual men attending a program for men who self-report problems with depression, isolation and social anxiety	51.2; men 100%	Intervention: Six sessions (6 consecutive weeks): (1) Identification of support network; (2) Activity scheduling and anticipating obstacles; (3) Problem-solving technique; (4) HIV/STI information; (5) Communication skills building Control: NA	Self-reported sexual behaviour in the past 30 days, condom use self-efficacy (CUSES), CES-D, SIAS, BFNE, UCLA loneliness scale	Immediate after intervention	84 (13%)	Complete case analysis; Linear regression adjusted for age and self-reported HIV status
Rose, 1996	IEC	USA, 1995	458	Older adults in 28 meal sites	75; women 75%	Intervention: One educational session of AIDS facts and prevention Control: NA	Self-reported HIV general knowledge, perceptions of susceptibility to AIDS, and perceptions of seriousness of AIDS, ten true-false statements on HIV knowledge	Immediate after intervention	318 (32%)	Complete case analysis; t-test
Saifu, 2011	Video	USA, 2010–11	150	Non-scheduled walk-in patients to a Veteran Affairs Clinic	60; men 98%	Intervention: Educational video (2 mins), with additional optional videos Control: Standard care	Uptake of HIV screening test	Immediate after intervention	NA	Intention to treat and complete case analysis; Logistic regression adjusted for age, gender and race
Saifu, 2011	Video	USA, 2010–11	44	Non-scheduled walk-in patients to a Veteran Affairs Clinic	58.9; men 100%	Intervention: Educational video (2 mins), with additional optional videos Control: Standard care	Self-reported HIV knowledge	Immediate after intervention	NA	Intention to treat and complete case analysis; Logistic regression adjusted for age, gender and race
Small, 2010	IEC	USA, 2007	50	People (aged 50+) living in Montgomery County	65 (median); men 68%	Intervention: One group educational session including facts about HIV and available services Control: NA	Self-reported: (1) HIV knowledge; (2) Interest in HIV education	Immediate after intervention	NA	Complete case analysis; ANOVA
**Qualitative**										
Gedin, 2014	IEC	USA	21	People (aged 55+) in low-income senior-housing communities	-	Intervention: One workshop of the Sexual Health for Older People program focusing on strengthening confidence and knowledge of sexual health Control: NA	Themes: (1) Utility of games through education (2) Positive impact of a well organised session (3) Importance of teach back approach (4) Timing is everything	Immediate after intervention	NA	Constant comparison analysis of data collected through focus group discussion
Reisner, 2011	IEC	USA, 2008–11	97	Older gay and bisexual men attending a program for men who self-report problems with depression, isolation and social anxiety	51.2; men 100%	Intervention: Six sessions (6 consecutive weeks): (1) Identification of support network; (2) Activity scheduling and anticipating obstacles; (3) Problem-solving technique; (4) HIV/STI information; (5) Communication skills building Control: NA	Unstructured written feedback from participants	Immediate after intervention	NA	None
Small, 2010	IEC	USA	50	People (aged 50+) living in Montgomery County	65 (median); men 68%	Intervention: One group educational session including facts about HIV and available services Control: NA	Themes: (1) Acknowledgement of risk-taking behaviours (2) Barriers to HIV education (3) Suggestions for implementation of HIV education programmes	Immediate after intervention	NA	Coding of data collected through focus group discussion

Abbreviations: IEC- information, education, and communication activity

The included studies covered a variety of study populations and settings. Four studies recruited participants from community-based settings [38, 39, 44, 46] while some focused on older people in retirement homes and senior centres (n = 1) [41], health and social services (n = 2) [43, 45]. The mean age ranged between 51 and 75 years old. Four studies only included men (n = 3) [38, 43, 45] or women (n = 1) [39], and one study focused on men who have sex with men [43], whilst one had a predominantly heterosexual sample [41]. The two RCTs included about 300 participants each [38, 39] while the sample sizes of quasi-experimental studies ranged between 40 [41] and 458 [44].

Information, education and communication activities (IECs, e.g. workshops) were the most common type of intervention (n = 7) in the included studies [38, 40–44, 46]. As defined by the World Health Organisation, IECs comprise activities which aim to “change or reinforce a set of behaviours in a ‘target audience’ regarding a specific problem in a predefined period of time” [47]. These aimed to foster participants’ knowledge on STIs and safer sex, with most specifically focusing on HIV. The length and frequency of IECs differed across studies. The interventions evaluated in five studies only had one-off sessions [18, 41, 42, 44, 46] while the interventions in two studies had multiple weekly sessions [40, 43]. In addition to a workshop, the study from China had a multicomponent intervention combining demonstration of condom use and sex education consultations over a one-year period [38]. Two studies from the US tested interventions other than IECs: one used a short educational video in a walk-in veteran clinic [45], and the other one designed website materials specifically for widowed or divorced women [39].

All the studies except for two [18, 39] focused on HIV prevention, rather than STIs in general.

Most studies used self-reported outcome measures and focused on knowledge (n = 6) [40–42, 44–46] and behavioural changes (n = 4) related to HIV [43], STIs [41] and safer sex [38, 39]. The only objective measure was uptake of HIV screening in a specific clinical setting [45]. Most outcomes (n = 5) were measured immediately after completion of the intervention [42–46]. Only two studies had additional follow-up at or after three months [40, 41]. The range of drop out or missing data was between 5% [38] and 88% [42]. However, all studies carried out complete case analysis. The analytical methods largely varied across studies, including simple testing of differences (chi-square test, t-test, paired t-test, ANOVA) (n = 4) [38, 41, 44, 46], ANCOVA (n = 1) [40], linear or logistic regression modelling with adjustment for covariates (n = 3) [39, 43, 45].

### Risk of bias assessment

**Fig 2:**
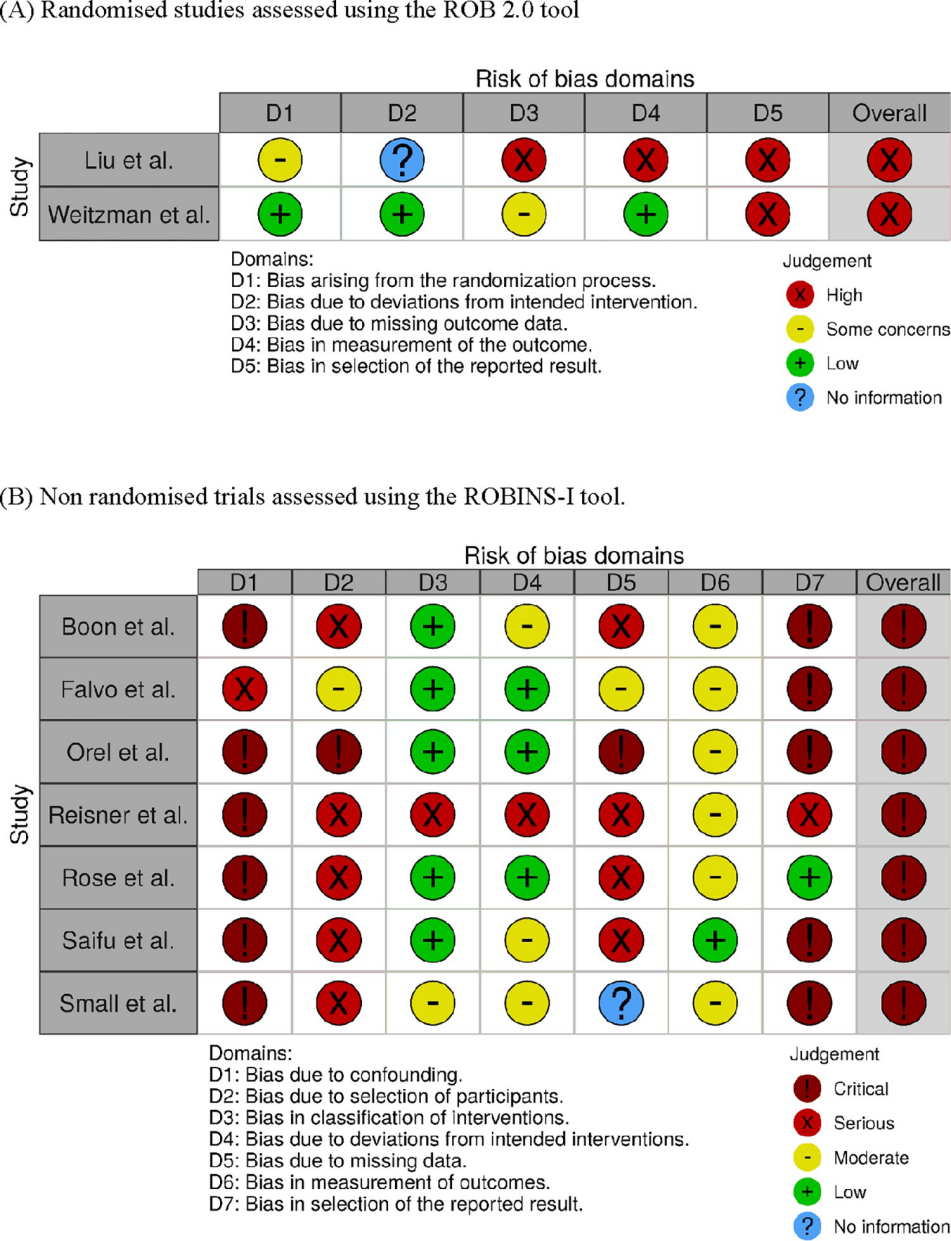
Risk of bias assessment. (A) Randomised studies assessed using the ROB 2.0 tool. (B) Non randomised trials assessed using the ROBINS-I tool.

The overall risk of bias across all studies was critical (Robins-I) or high (Rob-2.0) (Fig 2). Both randomised trials had a high risk of bias in the selection of the reported results, as there was no mention of a protocol or analyses being pre-specified in the protocol [38, 39]. The trial from Weitzman and colleagues had otherwise low risk or “some concerns” of bias in the other domains [39]. Liu et al’s study also had high risk of bias in other domains including the measurement of the outcome and missing outcome data [38]. All the pre-post studies had a critical risk of bias [40–46]. Bias due to the confounding was critical across the board (n = 7) [40–46]. Missing data were a problem across the studies (n = 6) [38, 40, 42–45]. Lack of control groups in quasi-experiment studies (n = 6) [40–44, 46] also affected the overall quality of the evidence. The risk of bias in measurement of outcomes was moderate to low in the non-randomised studies. Similarly, the risk of bias due to the classification of interventions was low in the non-randomised studies, with the exception of Small [46], which had a moderate risk of bias, and Reisner, O’Cleirigh [43] which had severe risk.

The included studies reporting qualitative outcomes shared a number of methodological issues, the most central being a lack of clarity in the description of the methods being used. This made it difficult to determine whether the methods for data collection, analysis or the conclusions drawn were appropriate.

### Effectiveness of non-pharmacological primary prevention interventions

The results of included studies are summarised in Table 2. In general, the included studies focused on two topics: HIV /STI and safer sex. The outcomes of interest were divided into three aspects: knowledge, behaviour change and other. Six studies focused on HIV /STI knowledge [40–42, 44–46]. Despite variation in length and frequency of IECs, four out of five studies suggested an improvement of HIV /STI knowledge after interventions across different study populations in the US and South Africa [40–42, 44]. However, it is unclear whether these improvements were retained over time. The study by Boon and colleagues suggested that the immediate improvement of knowledge after interventions might not be maintained after three months, whereas Falvo and Norman found that information was retained at the three-month follow-up [40, 41]. In addition to knowledge, one IEC intervention focusing on older US adults found increased perceived susceptibility and seriousness of HIV [44]. In a study carried out in a walk-in veteran clinic, a 2-minute educational video also was found to have a positive impact on HIV knowledge and interest in HIV education [45]. However, the video intervention did not increase uptake of HIV testing in the clinic.

**Table 2:** Summary of results of included studies.

Topic	Element	Results	References
HIV, STI	Knowledge	*IEC*: Positive effects on improvement of knowledge immediately after IEC sessions were found in four of the five studies (n = 5) *Educational video*: Positive effect on improvement of knowledge (n = 1)	Boon, 2009; Falvo, 2004; Orel, 2010; Rose, 1996; Small, 2010 Saifu, 2011
	Behavioural change	*Educational video*: No effect on increase in uptake of HIV testing (n = 1)	Saifu, 2011
	Other	*IEC*: Positive effect on perceived susceptibility and severity (n = 1) *Educational video*: Positive effect on increasing interests in HIV education (n = 1)	Rose, 1996 Saifu, 2011
Safer sex	Behavioural change	*IEC*: Limited effect on improving safer sex practice (n = 2) *Multiple components (workshop*, *demonstration and consulting)*: Positive effect on reducing high risk sex and increasing condom use (n = 1)	Reisner, 2011; Falvo, 2004 Liu, 2019
	Other	*Website materials*, *emails and texts*: positive effect on increasing intention to practice safer sex (n = 1) *IEC*: positive effect on increasing condom use self-efficacy (n = 1)	Weitzman, 2019 Reisner, 2011

Abbreviations: IEC- information, education, and communication activity

For safer sex practice outcomes, IEC interventions had limited effects on self-reported behavioural changes. One study focusing on older gay men only reported a positive effect of IEC interventions on increasing condom use self-efficacy yet no effects on reducing unprotected insertive anal or unprotected receptive anal episodes [43]. Researchers from another study, which measured past, present and future behaviours of vaginal and anal intercourse, oral sex and condom use, were unable to analyse the data as a large proportion of the participants refused to answer these questions [41]. Although the IEC approach did not lead to behavioural changes, the study focusing on older adults in China used a multicomponent intervention combining IECs, condom use demonstrations and sex education consultations over a one-year period. This study reported significant reductions in extra-marital sex and commercial exchange of sex for money, drug or gifts (although it is not clear whether this refers to the buying or selling (or both) of sex), and increased condom use in the intervention group compared to the control group without standard care [38]. Finally, a study that focused on widowed or divorced women designed an online intervention using website materials and frequent contacts by email and text [39]. The results showed a positive effect of the online intervention on enhancing self-reported safer sex intention compared to the control group who had only access to written materials.

### Barriers and facilitators to successful implementation

Three studies presented qualitative data on barriers and facilitators to implementing interventions [18, 43, 46]. The findings of the thematic synthesis are presented in Table 3. We identified two themes related to the barriers and facilitators to the successful implementation of non-pharmacological interventions and sexual education programmes in particular: “Perceived relevance of sexual education programmes”, and “Tailoring interventions for older adults”.

**Table 3:** Review of evidence from qualitative studies.

Review theme	Subtheme	Description	Evidence
“Perceived relevance of sexual education programmes”	N/A	There is a lack of appropriate sexual education programmes for older adults, due to widely held beliefs that they are not relevant to this age group. Effective sexual education programmes aim to address this perception.	Like I said, we’re the forgotten age group. And nobody feels like 50 and older is at risk because of the fact that we’re usually with the person that we’re with for so long that we’re not out here fooling around. Like I say, society just has to accept the fact that we’re still sexually active” (Small et al., 2010) This was actually the first thing I’ve seen like this—there’s not much available for guys in my age group—single guys in our forties." (Reisner et al., 2011) ‘Anything that anybody did when they were younger, selling them or buying, or anything else, they’re doing now. If they were promiscuous with sex, they’re doing it now. If they were able to get money on the side doing it, they’re doing it now. There’s always gonna be somebody willing to pay for it. [Group agrees]. (Small et al., 2010) ‘‘I think some of the people that I have ran into in my association over 50 and even above, more, they feel that they are more indefensible to the AIDS virus. That even in church the women are more unwilling to even want to discuss the risk of contracting AIDS.” (Small et al., 2010)
Tailoring interventions for older adults	Peer support	Older adults reported taboo attitudes being a barrier to attending sexual education programmes. Including elements of peer support or education was seen as a way of counteracting this.	‘‘I venture to say too there are people who don’t want to come because it’s taboo to them still, and it shouldn’t be, but maybe the person next door is peer pressuring. ‘You’re going to what?’ “(Small et al., 2010); ‘‘Or even has an older person infected with AIDS, show them that somebody older has this and how somebody older can get it.” (Small et al., 2010) "Meeting other people my age—it was kind of like finding out that you’re not the only one—there are other people in the same boat." (Reisner et al., 2011)
	Presentation of information in an engaging way	Participants enjoyed the inclusion of interactive elements. Some participants felt this may be helpful for people who struggle with memory.	‘‘Our memories aren’t real great at our age. I might remember something for the next week and then not think about it for about two weeks and then ‘Oh what was that about? Now I don’t remember.’ I want something I can refer to.”(Small, 2010) “The game really backed up our knowledge. It was a fun way to ask questions and reinforce knowledge.” (Gedin et al., 2014)
	Timing and location of the intervention	Timing and location is a big barrier for older adults taking part in sexual education interventions. Participants and researchers noted that interventions are more likely to be attended if they are situated within existing community services for older adults. However, participants did not agree on what would be the best time of day.	Participants felt that the timing and location of the intervention had the biggest impact on whether they, or their peers would attend. The suggested that sessions should be tied to other community programmes for older adults (Gedin et al., 2014); ‘‘I really think it needs to come to you where you live. It’s too hard to get out. I mean they’re not going to go out somewhere, most of us are not, and find out about things.” (Small et al. 2010)

### “Perceived relevance of sexual education programmes”

This theme describes the lack of availability of programmes that are relevant to the needs of older adults, some participants attributed this to societal beliefs about sexuality and ageing. However, they also reported that older adults may be reluctant to attend due to stigma or taboos surrounding sex.

One participant described the need for sexual education programmes that are relevant to their own circumstances: “This was actually the first thing I’ve seen like this—there’s not much available for guys in my age group—single guys in our forties." [43]. Participants reported being aware of societal beliefs that older adults are either in monogamous relationships or not sexually active, and therefore are not in need of sexual education programmes. “Like I said, we’re the forgotten age group. And nobody feels like 50 and older is at risk because of the fact that we’re usually with the person that we’re with for so long that we’re not out here fooling around. Like I say, society just has to accept the fact that we’re still sexually active’.” [46]. Participants reported that other people in their own age group may have similar beliefs regarding the relevance of sexual education programmes to people over the age of 50, which could be a barrier to attending such programmes. “I think some of the people that I have ran into in my association over 50 and even above, more, they feel that they are more indefensible to the AIDS virus.” [46].

Additionally, participants described sex as a taboo subject which may affect older adult’s willingness to participate in interventions focused on reducing STIs. The following theme describes how interventions could be tailored to highlight their relevance to and meet the needs of older adults.

### “Tailoring interventions for older adults”

This theme describes how interventions could be tailored for adults over the age of 50. Suggestions included: 1) including peer support 2) presenting information in an engaging way, and, 3) timing and location of the intervention.

1) Including peer support

Participants described the value of peer support for increasing attendance to sexual education programmes, creating an inclusive environment, and reinforcing the relevance of sexual education to their age group. "Meeting other people my age—it was kind of like finding out that you’re not the only one—there are other people in the same boat." [43]. Similarly, participants in another study described how the intervention could have been improved by the inclusion of a peer educator: “And just by one older person telling another older person about it, they’ll like it. Instead of them seeing a younger kid telling people.” [46].

2) Presentation of information in an engaging way

One study used games during their IEC session, where attendees taught skills and information they had learned back to the group. Participants described this as an engaging and “fun way to ask questions and reinforce knowledge” [18]. Participants reported these types of techniques may be helpful to some older adults whose “memories are not great” [46].

3) Timing and location of the intervention

One further consideration for the successful implementation of sexual education interventions is the location and timing of interventions. In one study, participants suggested that interventions should be locally available and could be anchored to existing community services or to programmes for older adults that are already running [18]. One participant summarised this by stating: “I really think it needs to come to you where you live. It’s too hard to get out.” [46]. However, Gedin and Resnick [18] reported that participants disagreed on which time of day was best with some preferring after 5pm and others expressing a preference for earlier times of the day.

## Discussion

### Summary and context

The interventions summarised in the present study were mainly IECs focusing on fostering participants’ knowledge of STIs and safer sex. Most of them specifically focused on HIV. The interventions generally reported an increase in STI/HIV knowledge. It is unclear to what extent this translates to behaviour changes in older adults. Furthermore, we cannot comment on whether these types of interventions can lead to a reduction in STIs as biological outcome measures were not used by the included studies. As well as knowledge of STIs, one study found that older adults’ perception of susceptibility to STIs increased, and this perception has been proposed as a necessary factor for behaviour change [48].

The qualitative analysis identified two themes related to the barriers and facilitators to the successful implementation of IECs for older adults. First, the perception that IEC are not relevant to adults over the age of 50 may contribute to the scarcity of interventions available. A successful IEC needs to address this perception and be aware that older adults may be reluctant to attend due to stigma and taboos surrounding sexuality and ageing. To address this perception, the intervention should be tailored to the needs of the group it is targeting. Some suggestions raised by the qualitative data included adding elements of peer support, presenting information in an engaging way and ensuring a convenient time and location for the interventions.

Our findings are similar to previous reviews on prevention of HIV in the older population, particularly as we found few recent studies on the topic (only three since 2011) and only two studies aiming to prevent STIs other than HIV [18, 39]. However, HIV prevention interventions may still promote safer sex practices that help to prevent other STIs as well. Previous reviews have also emphasised the lack of evidence-based, age-tailored interventions in the literature [7, 22, 23], and have reported ageism and lack of awareness of HIV in older adults [23], reflecting our theme: “the perceived relevance of sexual health interventions” to older adults”. Davis and Zanjani [22]’s review similarly found that studies reported improvements in HIV knowledge after primary prevention educational interventions.

While our review focused on primary prevention, interventions aimed at individuals with STIs have also been studied and are important in reaching older adults. Previous reviews have found that studies on behaviour change (such as increasing condom use and reducing sexual partners) have tended to focus on older people living with HIV, with mixed success [7, 22, 23]. Findings from evaluations and research on effective behaviour change interventions in other populations could potentially be adapted to target older adults without STIs as well. Reviews of studies carried out in younger individuals have shown that single-session interventions can lead to increased STI prevention, with minimal burden for both patients and providers [49]. However, a meta-analysis of trials of behaviour counselling interventions in adolescents found that longer contact interventions over two hours may have even greater effect than shorter lengths of contact time [50]. Similar to our findings, reviews in other adult populations have also suggested the effectiveness of culturally-tailored cognitive behavioural approaches for socioeconomically disadvantaged and minority ethnic women for knowledge, behaviour change and STI transmission outcomes [51] as well as short-term effectiveness of eHealth (including web or video based interventions similar to studies in our review, as well as other chat, multimedia, and social media interventions) for behaviour change outcomes in men who have sex with men [52]. Similar approaches may also benefit older populations if they are tailored to their context and needs.

Peer education and peer support, which were particularly suggested in qualitative studies in this review, have also been investigated in other adult populations in different settings with reported improvements in behavioural outcomes, such as knowledge of HIV, condom use, and uptake of testing services [53, 54], though clinic-based or clinician-led approaches have also been shown to be effective in other populations [50, 55]. However, other studies and reviews have echoed the difficulties of implementing and designing robust studies to evaluate such interventions, and particularly evaluating biological outcomes of STI infection [53, 56].

One of the most unfortunate results of our review, perhaps, is that limited evidence was found. This does not come as a surprise: a 2007 review of STI risk-reduction clinical trials already evidenced that over two-thirds of these actively excluded people over the age of 50 [57]. The situation does not seem to have changed much, with sexuality among older adults remaining largely unexplored and tainted by ageist perceptions in the media, among healthcare providers and researchers, and among both young and older adults [58]. Ageist perceptions and experiences may not only be limiting the reach of sexual health interventions on older adults, but may even have an impact on the sexual activity and interest of older adults [59]. Replacing these ageist perceptions of older adults’ sexuality in favour of more realistic ones remains a priority [58].

### Limitations

Studies covered a wide range of designs and outcomes. Many of the IECs were targeted at certain groups (e.g., specific genders or sexual orientations) and regions (US), making it difficult to generalise findings beyond those groups and locations. Participants were also often self-selected volunteers, who may not be representative of all older adults.

Strength of evidence in this review was low overall; only two studies had randomised controlled designs and the risk of bias was high across the studies, particularly due to confounding, participant selection methods, selection of reported results, and reporting of qualitative methods. While we excluded conference abstracts to ensure all studies included had undergone peer-review, it is possible that these may provide additional evidence and insight. We additionally did not contact study authors for further information, either for conference abstracts found or the articles included in this review.

Although most studies reported positive changes in knowledge of STIs and safer sex practices immediately after a behaviour change intervention, it is unclear from our review if this knowledge is retained over a longer period, as this was only measured in two studies with mixed results, and follow-up times were limited to 3 months or less. Particularly with pre-post designs, test-retest bias may inflate participants’ scores on post-intervention tests. Furthermore, changes in knowledge of STI can, but do not always, lead to changes in behaviour and transmission. It is important that future studies include both behavioural and biological outcome measures.

Reporting bias was possible with self-reported behavioural outcomes (e.g. STI testing, safer sex practices); in one study [41] participant’s reluctance to answer questions about sexual behaviours forced study authors to drop this as an outcome. Participants may not answer self-report questionnaires honestly for certain questions due to the stigma and taboo surrounding sexual behaviour, particularly in older adults. Multiple studies included [41, 42, 46] discussed how participants felt that sex was a taboo topic for their generation. Previous research has suggested that supporting participant’s privacy and anonymity can improve response rates to self-report measures [60]. Computer-based interviewing has been found to increase the reporting of sexual behaviours, compared self-report questionnaires [61]. However, more research is needed to explore the acceptability of computer-based measures among older adults.

We included a wide range of ages in our review, and participants may differ in acceptance and prior experiences of sexual education. In some studies, participants mentioned that their views around sex and sexual education were influenced by the attitudes of the time when they were growing up [41, 42]. There was not enough information for us to complete further subgroup analysis comparing younger and older adults within our age range. Additionally, all studies but the two RCTs were completed at least ten years ago. These results may be less generalisable to the older adults of today or of the future, who grew up in a different generation.

### Implications

Several key messages identified from the reviewed papers may help inform future interventions for STI prevention in older adults. The findings from the thematic synthesis indicate that sexual education interventions should be tailored to the needs of older adults. One barrier to participation for older adults is that they may not perceive such interventions as relevant to them. This is supported by Lewis et al. [19] who reported that middle and older-aged adults may feel disconnected from safer sex messages and services, which can be or seem targeted to younger people. One potential method that we identified for highlighting the relevance of sexual health interventions is to include elements of peer support. This is supported by existing evidence which suggests that face-to-face IEC interventions may have relevant positive characteristics, such as the presence of a peer group that may counterbalance the effect of stigma [42, 43, 46, 62]. Moreover, such interventions may be more widely accepted if educators are also older adults themselves. Training peers to provide education may increase accessibility and familiarity of interventions, and also provide role models closer to the participants’ characteristics [63].

Our qualitative findings also indicate that sexual health interventions should be presented in an informative and engaging way. While this may be helpful for some older adults, who may experience problems with memory functioning, it also applies to participants of any age. Moreover, educational content should be specifically tailored to the social and cultural context of the participants [64, 65]. The timing and location of the intervention may also be a barrier to attending sexual health interventions. Participants disagreed over what time of day would be best. This may be partly due to the wide range of ages in this review, where working age adults would prefer later times of the day and adults who were no longer working might prefer earlier times. Future researchers developing sexual health interventions should consider the specific needs of their target audience when deciding on the timing of the intervention. Some participants in the qualitative findings described wanting sexual health interventions to delivered locally and perhaps linked to existing community services, however they did not state which services they should be linked to. More research is needed to explore how the location of a sexual health intervention influences older adults’ likelihood of attending.

There is a limited range of interventions being tested in this area. Seven of the 10 interventions included in this review were IECs. Other intervention types may also be useful: website materials, emails and texts, and educational videos enhanced safer sex intentions in our review [39, 45]. Such interventions have been found useful in younger adults, including digital programmes aimed at increasing condom use in men, or information helplines to provide information about HIV to the population [66, 67]. Similar interventions may be useful for older adults as well; a survey of older adults in Australia suggested that the internet was a primary source of information about sex for many respondents, and the rise of social media and dating apps in older adult populations may present another medium by which to provide STI education [68, 69]. Digital interventions offer several potential advantages to in-person IECs. They can help maintain participant privacy and anonymity, be used to deliver individualised information, and may be cheaper to deliver [25]. These digital interventions could also be embedded into larger multi-component programmes, in addition to more traditional face to face IECs. However, accessibility should be carefully considered for populations that are economically disadvantaged or have low digital access or literacy. Half of the interventions included in this review were one-off events. Future research is needed to explore whether increasing the number of sessions in the intervention was associated with increased effectiveness. Furthermore, participants should be follow-up for longer to determine whether these interventions lead to lasting changes.

It is therefore important that researchers develop context-tailored interventions that address the educational needs of older adults, and test them using large and well-conducted RCTs, with pre-specified protocols and analyses. Only then we will be able to assess the effectiveness and benefits of those interventions. Future research should examine how best to deliver sexual health interventions in this population, including services for both STI prevention as well as treatment.

## Conclusion

Literature on educational interventions for older adults is sparse, particularly outside the US and for STIs other than HIV. There is evidence that IECs may improve short-term knowledge about STIs, but less evidence for long-term improvement or behaviour change. More robust and higher-quality studies are needed in order to confirm the effectiveness of behaviour change interventions, and on the whole, more research is needed to understand and address the particular sexual health issues faced by older adults.

## Supporting information

S1 Appendix PRISMA checklist. (DOCX)

S2 Appendix Search strategy. (DOCX)

S1 Data (XLSX)
