# Supplementary material for: Non-pharmacological interventions for the prevention of sexually transmitted infections (STIs) in older adults: A systematic review
Source: PLoS One. 2023 May 24;18(5):e0284324. doi: 10.1371/journal.pone.0284324 (PMC10208510; doi:10.1371/journal.pone.0284324)
Supplement: S2 Appendix — (DOCX) [file pone.0284324.s002.docx]

### ***S2 Appendix: Search strategy***

The following search strategy was used in EMBASE, MEDLINE, PSYCINFO AND GLOBAL HEALTH:

1. (STI or STD or VD or sexually transmitted or sexually-transmitted or venereal disease).mp. [mp=ti, ab, hw, tn, ot, dm, mf, dv, kw, fx, dq, nm, kf, ox, px, rx, an, ui, sy, bt, id, cc, tc, tm, sh, mh]

2. exp Sexually Transmitted Diseases/

3. 1 or 2

4. (older adult* or elder* or geriatric or late?-life or ag?ing).mp. [mp=ti, ab, hw, tn, ot, dm, mf, dv, kw, fx, dq, nm, kf, ox, px, rx, an, ui, sy, bt, id, cc, tc, tm, sh, mh]

5. exp Aged/

6. exp Geriatrics/

7. 4 or 5 or 6

8. (prevent* or intervention or educat* or behavio?r* or communicat* or counsel*).mp. [mp=ti, ab, hw, tn, ot, dm, mf, dv, kw, fx, dq, nm, kf, ox, px, rx, an, ui, sy, bt, id, cc, tc, tm, sh, mh]

9. exp Health Education/

10. 8 or 9

11. (contracept* or condom or risky sex* or safe sex* or unsafe sex*).mp. [mp=ti, ab, hw, tn, ot, dm, mf, dv, kw, fx, dq, nm, kf, ox, px, rx, an, ui, sy, bt, id, cc, tc, tm, sh, mh]

12. exp Condoms, Female/ or exp Contraception/ or exp Condoms/ or exp Contraception Behavior/

13. 3 or 11

18. 7 and 10 and 13

The following search was carried out in the Cochrane Library

1. MeSH descriptor: [Aged] explode all trees
2. MeSH descriptor: [Sexually Transmitted Diseases]
3. 1 and 2
